# Cardioprotective effects of naringin, hesperidin, and hesperetin: modulation of atherosclerosis, cardiac remodeling, and myocardial ischemia/infarction

**DOI:** 10.3389/fnut.2026.1742472

**Published:** 2026-05-26

**Authors:** Yuzhang Sun, Zhejun Zhao, Yuanlong Sun, Xuyuan Lao, Naiwen Chen, Zhaofeng Shi, Shaohui Wu, Xiaofen Ruan

**Affiliations:** 1 Cardiovascular Research Institute, Shanghai Shuguang Hospital Affiliated to University of Traditional Chinese Medicine, Shanghai, China; 2 Department of Urology, Shuguang Hospital Affiliated to Shanghai University of Traditional Chinese Medicine, Shanghai, China; 3 Department of Cardiology, Shanghai Chest Hospital, School of Medicine, Shanghai Jiao Tong University, Shanghai, China

**Keywords:** anti-atherosclerotic effects, cardiovascular diseases, hesperetin, hesperidin, naringin

## Abstract

Cardiovascular diseases (CVDs) persist as the predominant contributor to morbidity and mortality on a global scale, principally attributed to the pathophysiological processes of atherosclerosis, cardiac remodeling, and myocardial ischemia/infarction. Contemporary research underscores the cardioprotective efficacy of natural flavonoids, including naringin, hesperidin (HES), and hesperetin (HST), bioactive constituents prevalent in citrus fruits. These flavonoids demonstrate multifaceted effects, encompassing antioxidant, anti-inflammatory, anti-apoptotic, lipid-lowering, and endothelial-protective properties, all of which synergistically enhance cardiovascular performance. At the molecular nexus, naringin, HES, and HST influence critical signaling pathways, thus diminishing oxidative stress, obstructing the action of inflammatory cytokines, and curtailing the proliferation of vascular smooth muscle cells. Furthermore, these bioactive compounds regulate cardiac remodeling by alleviating fibrosis, hypertrophy, and mitochondrial dysfunction, whilst simultaneously promoting angiogenesis and optimizing energy metabolism. In experimental models of myocardial ischemia and infarction, they facilitate cardiomyocyte viability and diminish infarct size through the modulation of apoptosis-related genes and the mitigation of oxidative injury. This review meticulously synthesizes the mechanistic insights and preclinical data that substantiate the cardioprotective properties of naringin, HES, and HST. Additionally, it examines their pharmacokinetic profiles, bioavailability, and prospective clinical applications as adjunctive agents in the prophylaxis and management of CVDs. Elucidating these molecular pathways could offer a promising framework for the formulation of predictive, preventive, and personalized strategies aimed at combatting CVDs.

## 1 Introduction

Cardiovascular diseases (CVDs) persist as the predominant cause of both mortality and morbidity on a global scale, accounting for approximately 19 million fatalities each year and serving as the principal contributor to worldwide disability-adjusted life years (1, 2). Notwithstanding advancements in pharmacotherapeutic and interventional modalities, the absolute prevalence of CVD continues to escalate, driven by demographic aging, alterations in lifestyle, and the worldwide surge in metabolic disorders (3). Atherosclerosis, cardiac remodeling, and myocardial ischemia/infarction constitute the principal pathophysiological mechanisms that underlie the majority of cardiovascular incidents (4, 5). Consequently, the identification of cost-effective, preventive, and adjunctive therapeutic strategies remains a paramount priority in the realm of global health.

In contemporary research, focus has increasingly shifted toward dietary bioactive compounds due to their potential role in cardiovascular protection (6, 7). Flavanones, a specific subclass of flavonoids primarily found in citrus fruits, have surfaced as promising therapeutic agents owing to their antioxidant, anti-inflammatory, lipid-lowering, and vasoprotective attributes (8, 9). Epidemiological investigations have indicated a negative correlation between the intake of citrus fruits and the risk of CVDs, implying that diets rich in flavanones may play a role in the prophylaxis of CVDs (10–12). Among the various flavanones, naringin (NRG), hesperidin (HES), and hesperetin (HST) have garnered significant attention regarding their effects on cardiovascular health. Naringin, which is predominantly located in grapefruits, along with HES, which is prevalent in oranges and lemons, are glycosides that are converted *in vivo* into their corresponding aglycones, namely naringenin and HST (13, 14). These compounds are structurally characterized by a flavanone backbone, exhibiting variations in hydroxyl and methoxy substituents as well as glycosylation patterns that critically affect their solubility and bioavailability (15). Despite the limited intestinal absorption associated with their glycosidic forms (naringin, HES), the processes of microbial enzymatic hydrolysis and phase II conjugation reactions significantly enhance systemic availability of their bioactive metabolites (16, 17). Innovative approaches, including nanoformulation and co-administration with bioenhancers, are currently being explored to optimize their pharmacokinetic characteristics (18–20).

Experimental and preclinical investigations provide compelling evidence for the cardioprotective efficacy of these flavanones via the modulation of diverse molecular pathways. Naringin and HES have been shown to diminish low-density lipoprotein oxidation, mitigate endothelial dysfunction, inhibit the proliferation of vascular smooth muscle cells, and reduce the expression of inflammatory cytokines which are critical mechanisms implicated in the onset and advancement of atherosclerosis (21). Moreover, both compounds exhibit notable anti-fibrotic and anti-hypertrophic properties, thereby attenuating cardiac remodeling through the regulation of TGF-β, NF-κB, and MAPK signaling cascades (22, 23). In experimental models of myocardial ischemia and reperfusion injury, HES and naringin confer protection to cardiomyocytes by scavenging reactive oxygen species, preserving mitochondrial integrity, and inhibiting apoptotic signaling pathways (24, 25). HST, the aglycone metabolite, demonstrates robust antioxidant and anti-apoptotic capabilities while enhancing myocardial function subsequent to ischemic injury (22, 26). Notwithstanding the persuasive preclinical evidence, the translational applicability of these flavanones in the context of human cardiovascular health remains inadequately defined. Constraints include varying bioavailability, inconsistencies in dose–response relationships, and a paucity of clinical trial data (10). Consequently, it is imperative to systematically synthesize the extant evidence to clarify the cardioprotective mechanisms associated with naringin, HES, and HST, as well as to investigate their therapeutic potential in the realm of CVDs.

Several recent reviews have discussed the biological activities of citrus flavonoids, primarily emphasizing antioxidant and anti-inflammatory properties or focusing on single compounds such as hesperidin or hesperetin. However, these reports often address isolated mechanistic aspects or a single cardiovascular condition, most commonly atherosclerosis. The present review differs in several important respects. First, it provides a comparative and integrative analysis of NRG, HES, HST within a unified cardiovascular framework. Second, it systematically examines their roles across multiple interconnected pathological processes, including atherosclerosis, cardiac remodeling, myocardial ischemia/reperfusion injury, and myocardial infarction. Third, it incorporates emerging mechanistic insights, such as ferroptosis regulation, gut microbiota–cholesterol axis modulation, and microRNA-dependent pathways, which have not been comprehensively synthesized in prior literature. Finally, this review critically addresses pharmacokinetic limitations, bioavailability challenges, and translational barriers, thereby providing a more comprehensive perspective that bridges mechanistic evidence with clinical applicability.

In light of this, the present review intends to thoroughly examine the molecular mechanisms and empirical findings that support the cardioprotective effects of these flavanones in the contexts of atherosclerosis, cardiac remodeling, and myocardial ischemia/infarction, thereby underscoring their potential clinical significance and translational hurdles.

## 2 Chemical structure, sources, and bioavailability

**Figure 1:**
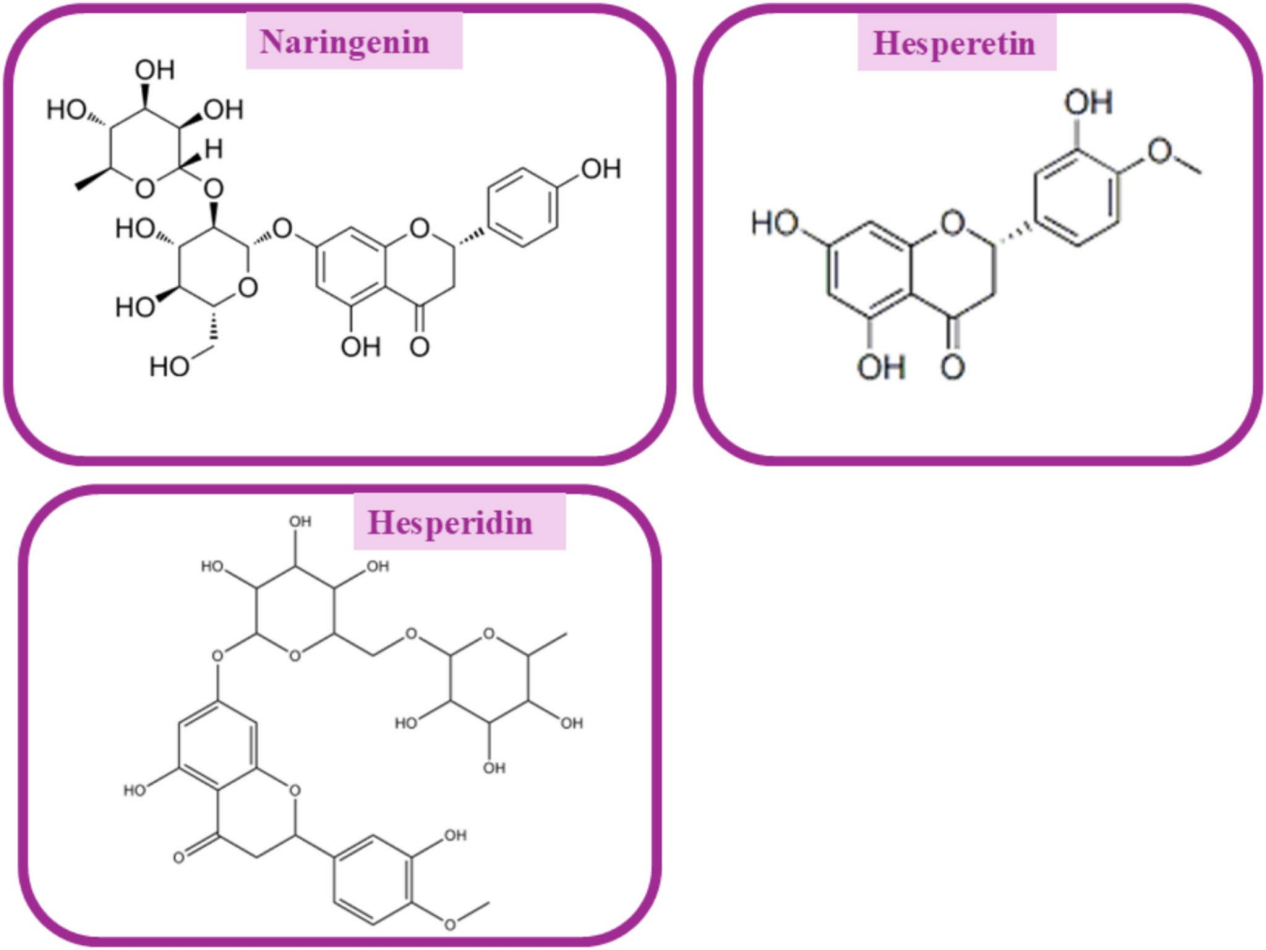
Standardized chemical structures of naringin, hesperidin, and hesperetin.

Naringin, HES, and HST are classified within the flavanone subclass of flavonoids, which are polyphenolic entities defined by the C6–C3–C6 carbon framework composed of two aromatic rings (A and B) interconnected by a heterocyclic pyran ring (C). Flavanones are distinguished from other flavonoid subclasses (e.g., flavones, flavonols, isoflavones) by the presence of a saturated C2–C3 bond and a carbonyl functional group at position C4 within the C-ring, imparting unique biochemical characteristics (Figure 1) (27). Naringin is identified as the 7-O-neohesperidoside derivative of naringenin (4′,5,7-trihydroxyflavanone), whereas HES is recognized as the 7-O-rutinoside variant of HST (3′,5,7-trihydroxy-4′-methoxyflavanone) (Figure 1) (13, 28). The corresponding aglycones of naringenin and HST are devoid of the sugar moiety, enhancing their lipophilicity and facilitating superior absorption across intestinal membranes (15). The presence or absence of glycosidic bonds, along with the specific nature of the attached sugar residues, plays a critical role in determining solubility, intestinal transport mechanisms, and metabolic pathways (16).

The dietary origins of these flavanones are principally derived from citrus fruits and their respective derivatives. Naringin is particularly prevalent in grapefruits (Citrus paradisi) and pomelos (*Citrus maxima*), contributing to their distinctive bitter taste (8). HES is chiefly located in sweet oranges (*Citrus sinensis*), lemons (*Citrus limon*), and tangerines (*Citrus reticulata*), with a notable concentration in the peel and albedo (13, 15). HST, which serves as the aglycone of HES, is infrequently encountered in its free form within natural sources; however, it is synthesized during intestinal metabolism or extracted for incorporation into dietary supplements and nutraceutical products (28). Commercial formulations of citrus flavonoids are extensively promoted as supplements with cardioprotective, antioxidant, and anti-inflammatory properties.

The bioavailability of flavanones serves as a fundamental factor influencing their biological effectiveness. Naringin and HES exhibit suboptimal absorption in their native glycosidic configurations due to their elevated molecular weight and hydrophilic nature (15, 17). Following ingestion, these compounds primarily arrive at the small intestine in an unaltered state, where they undergo enzymatic hydrolysis facilitated by β-glucosidases or intestinal microbiota, resulting in the formation of their respective aglycones of naringenin and HST which possess enhanced absorption characteristics (16, 17). Subsequent to absorption, aglycones are subjected to phase II metabolic processes within enterocytes and the liver, leading to the production of glucuronidated, sulfated, or methylated conjugates that are transported in the plasma (15). These metabolites have the capacity to be deconjugated in peripheral tissues, thereby establishing a persistent reservoir of bioactive entities (8). Pharmacokinetic investigations elucidate that subsequent to oral administration, peak plasma concentrations of HST and naringenin are observed approximately 4–7 h following ingestion (10). Nevertheless, their absolute bioavailability remains inferior to 20%, primarily attributable to inadequate intestinal absorption, first-pass metabolism, and efflux mediated by transporters such as P-glycoprotein (28, 29). Determinants affecting their bioavailability encompass food matrix composition, diversity of intestinal microbiota, activity of conjugating enzymes, and genetic polymorphisms within metabolic enzymes (30, 31). Initiatives aimed at augmenting systemic exposure have concentrated on nanoencapsulation, liposomal delivery systems, concomitant administration with piperine, and phospholipid complexes, all of which enhance stability and intestinal absorption (32, 33). Recent advancements in formulation technologies have demonstrated promising outcomes in improving oral bioavailability and tissue distribution, thereby amplifying their therapeutic efficacy for cardiovascular protection (34, 35). In summary, the compositional heterogeneity, nutritional richness, and adjustable pharmacokinetic characteristics of naringin, HES, and HST render them compelling candidates for both nutritional and therapeutic interventions in the prevention and management of CVDs. However, the variability in absorption and metabolism among individuals constitutes a significant obstacle that necessitates further exploration through pharmacogenomic and clinical research.

## 3 Anti-atherosclerotic effects of naringin, HES, and HST

Flavonoids including naringin, HES, and HST demonstrate significant anti-atherosclerotic properties through various molecular pathways, encompassing antioxidant, anti-inflammatory, lipid-regulating, and endothelial-protective effects (36–38). Naringin, a predominant flavanone glycoside found in grapefruit and other citrus species, has been evidenced to decrease serum levels of total cholesterol, triglycerides, and low-density lipoprotein cholesterol (LDL-C), while concurrently increasing high-density lipoprotein cholesterol (HDL-C) (39). It inhibits lipid accumulation within macrophages and obstructs the formation of foam cells. HES, another prevalent flavonoid derived from citrus, manifests robust vasculoprotective and lipid-modulating characteristics. HES has been shown to reduce levels of cholesterol and triglycerides, while concurrently enhancing endothelial function through the augmentation of nitric oxide bioavailability via the activation of endothelial nitric oxide synthase (eNOS) (40). Additionally, HES mitigates oxidative stress by promoting the activities of antioxidant enzymes such as superoxide dismutase (SOD), catalase (CAT), and glutathione peroxidase (GPx), and it inhibits the production of inflammatory cytokines, including tumor necrosis factor-alpha (TNF-α), interleukin-6 (IL-6), and C-reactive protein (CRP), all of which are instrumental in the pathogenesis of atherosclerotic plaque formation (41). In experimental animal models of atherosclerosis, the administration of HES resulted in a significant reduction in aortic plaque area and an enhancement of the histological characteristics indicative of vascular integrity (42). HST exhibits superior bioavailability and manifests analogous, albeit frequently more pronounced, biological effects (43). Furthermore, HST augments cholesterol efflux by upregulating ATP-binding cassette transporter A1 (ABCA1) and ATP-binding cassette transporter G1 (ABCG1), while simultaneously downregulating hydroxymethylglutaryl-CoA reductase, the pivotal enzyme regulating cholesterol biosynthesis (44). The aforementioned effects collectively facilitate the attenuation of atherosclerotic lesion progression and the enhancement of vascular functionality. In summary, these results indicate that naringin, HES, and HST may serve as promising phytochemical agents for the prophylaxis and management of atherosclerosis by modulating various molecular pathways associated with lipid metabolism, oxidative stress, and vascular inflammation (45).

### 3.1 Studies investigating the anti-atherosclerotic effects of naringin

**Figure 2:**
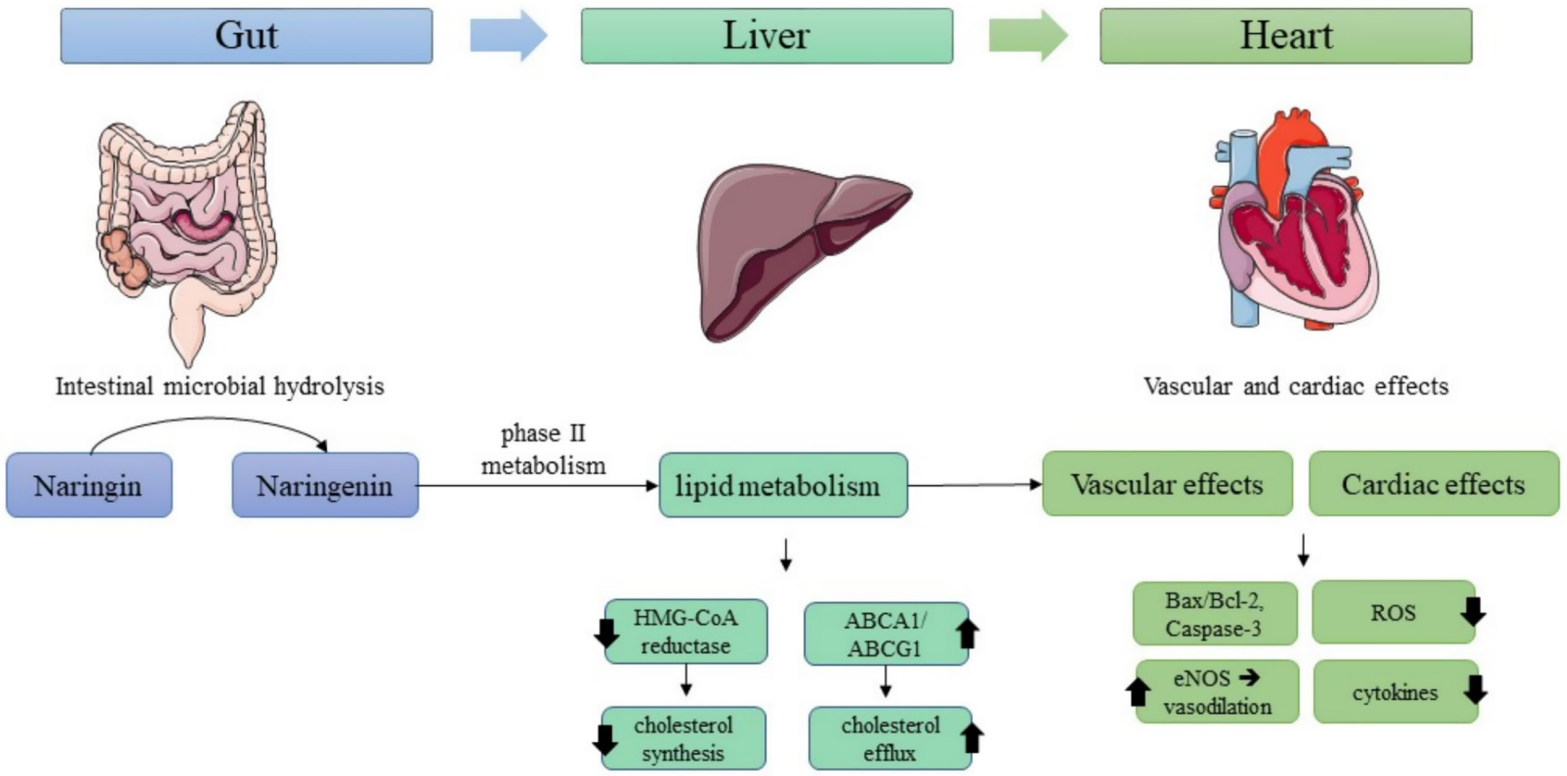
Naringin modulates the gut–liver–heart axis to exert cardioprotective effects. Naringin from citrus fruits is hydrolyzed by gut microbiota to naringenin, which enhances lipid metabolism and antioxidant capacity. Through activation of PI3K/Akt and β-catenin signaling and inhibition of NF-κB and MAPK pathways, naringin promotes cholesterol efflux (↑ABCA1/ABCG1), reduces HMG-CoA reductase activity, and protects cardiomyocytes by lowering ROS generation and apoptosis while improving endothelial nitric-oxide synthase (eNOS) activity.

Atherosclerosis continues to be a predominant contributor to cardiovascular morbidity and mortality on a global scale, propelled by factors such as lipid accumulation, oxidative stress, and persistent vascular inflammation. In light of the drawbacks and adverse effects associated with conventional lipid-lowering interventions, there is an augmented interest in the identification of natural compounds exhibiting multi-targeted protective effects. Naringin, a flavonoid derived from citrus fruits, has emerged as a noteworthy bioactive compound possessing lipid-lowering, antioxidant, and anti-inflammatory characteristics that effectively mitigate atherosclerotic advancement (Figure 2). Atherosclerosis arises as a consequence of vascular injury and oxidative stress, with the protection afforded by blood cells serving a crucial preventive function. An investigation explored the anti-atherosclerotic efficacy of naringenin along with its derivatives, naringin and naringin dihydrochalcone, concentrating on their impacts on erythrocytes, peripheral blood mononuclear cells (PBMCs), and platelets. All compounds conferred protection to erythrocytes against oxidative damage without compromising cellular membranes or influencing the metabolic processes of PBMCs and platelets. Interestingly, naringin and naringin dihydrochalcone exhibited a more pronounced inhibitory effect on collagen-induced platelet aggregation in comparison to naringenin. Although none induced liposome aggregation, naringenin and naringin dihydrochalcone did modify the dipole potential of liposomes. Ultimately, naringin dihydrochalcone demonstrated marginally enhanced protective activity, indicating its promising potential for the development of anti-atherosclerotic therapies (38) (Table 1).

**Table 1:** Summary of studies investigating the anti-atherosclerotic effects of naringin, hesperidin, and hesperetin.

Compound(s)	Experimental model/approach	Intervention duration	Main findings	Proposed mechanisms	Reference
Naringin, naringenin, naringin dihydrochalcone	*In vitro* (erythrocytes, PBMCs, platelets)	-	All flavonoids protected blood cells from oxidative damage; naringin and dihydrochalcone inhibited platelet aggregation	Antioxidant activity; inhibition of platelet aggregation	(38)
Naringin, naringenin, hesperidin, hesperetin	ApoE−/− mice (in vivo)	8–12 weeks	Naringin showed strongest anti-atherogenic effect (55.9% plaque reduction); others less potent	Gut microbiota–FXR/FGF15–CYP7A1 pathway; cholesterol metabolism regulation	(46)
Naringin and other flavonoids	*In vitro AS model using aortic vascular rings*	48 h	Naringin and related flavonoids inhibited VEGF, CRP, JNK2, and p38 expression	Anti-inflammatory effects via MAPK pathway inhibition	(49)
Naringin	HUVECs (*in vitro*)	12–24 h	Naringin inhibited TNF-α–induced VCAM-1, ICAM-1, E-selectin, CX3CL1, MCP-1, and RANTES	Suppression of IKK/NF-κB activation	(50)
Naringenin and quercetin	Network pharmacology + *in vitro* (RAW264.7)	24–48 h	Both compounds suppressed IL-1β and MMP9; identified as main bioactive ingredients	IL-1β/MMP9 pathway inhibition; anti-inflammatory and antioxidant effects	(51)
Naringin	ApoE−/− mice (*in vivo*)	8–12 weeks	Reduced serum and liver cholesterol; promoted bile acid excretion; improved gut microbiota composition	FXR/FGF15–CYP7A1 axis; enhanced cholesterol catabolism and reverse transport	(52)
Hesperidin	*In vitro* LDL and HDL oxidation assays	2–6 h	Hesperidin and luteolin inhibited LDL/HDL oxidation and apoA-I aggregation	Antioxidant activity; inhibition of lipid peroxidation and protein carbonylation	(54)
Hesperidin	LDLr−/− mice (*in vivo*)	8–12 weeks	Hesperidin ameliorated hyperlipidemia, reduced plaque area, improved insulin resistance	Regulation of ABCA1/G1/G8; suppression of ACCα and FAS; antioxidant and anti-inflammatory actions	(42)
Hesperidin	ApoE−/− mice (*in vivo*)	8–12 weeks	Reduced plaque formation, oxidative stress, and inflammation; improved gut microbiota composition	Modulation of gut microbiota–BCAA–host metabolic axis	(55)
Hesperidin	ApoE−/− mice + RAW 264.7 macrophages	-	Hesperidin prevented varenicline-induced increase in plaque size	Inhibition of CD36 and LOX-1; upregulation of ABCA1 and ABCG1; reduced oxLDL uptake	(56)
Hesperidin, hesperetin, quercetin, rutin	Hypercholesterolemic rabbits (*in vivo*)		Extract rich in hesperidin-like flavonoids reduced LDL, triglycerides; increased HDL and antioxidant enzymes	Antioxidant and lipid-lowering activity; inhibition of plaque formation	(57)

ABCA1, ATP-binding cassette transporter A1; ABCG1, ATP-binding cassette transporter G1; ACCα, Acetyl-CoA carboxylase alpha; ApoE−/−, Apolipoprotein E knockout mouse; BCAA, branched-chain amino acids; CD36, cluster of differentiation 36 (scavenger receptor); CRP, C-reactive protein; CYP7A1, cholesterol 7 alpha-hydroxylase; FAS, fatty acid synthase; FGF15, fibroblast growth factor 15; FXR, farnesoid X receptor; HDL, high-density lipoprotein; HUVEC, human umbilical vein endothelial cell; ICAM-1, intercellular adhesion molecule 1; IL-1β, interleukin-1 beta; IKK, IκB kinase; JNK2, c-Jun N-terminal kinase 2; LDL, low-density lipoprotein; LDLr−/−, Low-density lipoprotein receptor knockout mouse; LOX-1, lectin-like oxidized LDL receptor-1; MAPK, mitogen-activated protein kinase; MCP-1, monocyte chemoattractant protein-1; MMP9, matrix metalloproteinase-9; NF-κB, nuclear factor kappa-light-chain-enhancer of activated B cells; PBMC, peripheral blood mononuclear cell; RANTES, regulated on activation, normal T cell expressed and secreted (CCL5); TNF-α, tumor necrosis factor-alpha; VCAM-1, vascular cell adhesion molecule 1; VEGF, vascular endothelial growth factor.

In a comparative investigation utilizing ApoE^−^/^−^ murine models, naringin displayed the most pronounced anti-atherogenic effect, diminishing atherosclerotic plaque formation by 55.92%, followed sequentially by naringenin (42.87%), HES (34.98%), and HST (24.70%). Naringin primarily functioned within the intestinal environment, where it augmented bile acid synthesis via the gut microbiota–FXR/FGF15–CYP7A1 signaling pathway. Conversely, HES, naringenin, and HST primarily exerted their effects within hepatic tissues. HES facilitated cholesterol efflux through the upregulation of ABCA1, while naringenin and HST impeded cholesterol synthesis by downregulating HMGCR. The reduced absorption of HST, attributable to its 4′-methoxyl group, elucidated its diminished efficacy. Collectively, variances in metabolic processes and signaling cascades elucidated the divergent anti-atherosclerotic activities of these flavanones, thereby providing valuable insights for the formulation of flavonoid-based preventative strategies against atherosclerosis (46).

A cost-efficient and effective model for atherosclerosis was developed utilizing vascular rings from the thoracic aorta, assessed through vascular morphology, smooth muscle cell proliferation, and the quantification of inflammatory markers including VEGF, CRP, JNK2, and p38 (47). Employing this model, the anti-atherosclerotic properties of seven flavonoids, narirutin, naringin, eriodictyol, luteolin, galuteolin, astragalin, and kaempferol, extracted from Helichrysum arenarium L. Moench were systematically examined (47). All compounds demonstrated an inhibitory effect on the expression of inflammatory mediators and diminished the production of nitric oxide (NO) in LPS-stimulated macrophages (48). Comparative assessments indicated that flavonol aglycones exhibited superior anti-inflammatory and anti-atherosclerotic efficacy relative to their glycosides, with flavonols presenting greater potency than both flavanones and flavones. From a mechanistic perspective, it is posited that these flavonoids may attenuate the progression of atherosclerosis by downregulating CRP and inhibiting the activities of JNK2 and p38 kinases, consequently obstructing the MAPK signaling pathway, reducing NO and VEGF levels, and averting vascular stenosis and scar tissue formation. These results underscore the therapeutic promise of flavonoids in the prophylaxis of atherosclerosis (49). Another work examined the impact of naringin on TNF-α–induced inflammatory processes within human umbilical vein endothelial cells. Naringin demonstrated a dose-dependent efficacy in inhibiting THP-1 monocyte adherence to TNF-α-stimulated endothelial cells and significantly reduced the mRNA and protein levels of cell adhesion molecules (VCAM-1, ICAM-1, and E-selectin) alongside chemokines (fractalkine/CX3CL1, MCP-1, and RANTES). From a mechanistic standpoint, naringin interfered with the NF-κB signaling cascade by obstructing the phosphorylation of IKKα/β, IκB-α, and NF-κB, thus averting the nuclear translocation of NF-κB. These results exhibited that naringin mediates its anti-atherosclerotic properties predominantly through the attenuation of TNF-α–induced endothelial inflammation via the inhibition of the IKK/NF-κB signaling pathway (50).

An investigation examined the bioactive constituents and molecular mechanisms associated with Folium Artemisiae argyi in the management of atherosclerosis (AS) through the application of systems pharmacology and molecular docking methodologies. Through comprehensive database analyses, eight pivotal compounds and 232 prospective target genes were discerned, with quercetin and naringenin identified as principal active agents (51). Significant target genes encompassed VEGFA, MMP9, and IL-1β, which are intricately related to vascular inflammation and remodeling processes. KEGG pathway analysis indicated that the pathway of fluid shear stress and atherosclerosis is integral to the herb’s anti-atherosclerotic properties. Validation of gene expression revealed a downregulation of VEGFA, whereas MMP9 and IL-1β exhibited upregulation in patients with atherosclerosis. Molecular docking analyses illustrated a robust binding affinity of quercetin to MMP9, and *in vitro* cellular experiments indicated that both quercetin and naringenin substantially attenuated MMP9 expression and inflammatory responses. Collectively, Folium Artemisiae argyi demonstrates considerable anti-inflammatory and anti-atherosclerotic efficacy (51). Naringin demonstrates significant potential in the prophylaxis of atherosclerosis through the modulation of the gut microbiota–liver–cholesterol axis (52). In ApoE^−^/^−^ murine models subjected to a high-fat diet, naringin markedly diminished serum and hepatic cholesterol concentrations by 24.04 and 28.37%, respectively, and mitigated the progression of atherosclerotic lesions. Metabolomic analyses indicated that naringin induced alterations in hepatic cholesterol metabolites and bile acids, resulting in a 1.6-fold and 4.3-fold increase in bile acid and neutral sterol excretion, respectively (52). From a mechanistic perspective, naringin facilitated the biosynthesis of bile acids from cholesterol by upregulating CYP7A1 through the inhibition of the FXR/FGF15 signaling pathway and modulated gut microbiota that produces bile salt hydrolase and 7α-dehydroxylase. Furthermore, it augmented reverse cholesterol transport by downregulating PCSK9/IDOL (52). These findings underscored the regulatory function of naringin in cholesterol metabolism via the remodeling of gut microbiota, thereby providing a mechanistic framework for the formulation of functional foods aimed at the prevention of atherosclerosis (52). Overall, naringin exhibits considerable potential as a natural therapeutic agent against atherosclerosis through its modulation of lipid metabolism, attenuation of oxidative stress, and inhibition of vascular inflammation. Its multi-targeted influences on crucial molecular pathways, including PPARγ, LXRα, and Nrf2, underscore its potential for the prevention and management of CVDs. Subsequent clinical and translational investigations are imperative to validate its efficacy, enhance bioavailability, and examine its prospective role in personalized medicine strategies.

### 3.2 Studies investigating the anti-atherosclerotic effects of HES, and HST

The oxidation of LDL-C and HDL-C serves as a significant factor in the etiology of atherosclerosis (53).

One investigation assessed the inhibitory effects of the fruit extract of *Vitex rotundifolia*, as well as its six isolated constituents, artemetin, casticin, HES, luteolin, vitexin, and vanillic acid, on the oxidation processes of LDL-C and HDL-C. Employing biochemical assays that evaluated lipid peroxidation, conjugated dienes, carbonyl content, and apoA-I aggregation, the extract and notably the compounds casticin (2) and luteolin (4) exhibited pronounced inhibition of both LDL-C and HDL-C oxidation (54). These compounds led to a decrease in lipid peroxidation, a reduction in negative charge and hyperchromicity, and inhibited apoA-I aggregation. The findings demonstrated that casticin and luteolin possess significant anti-oxidative and anti-atherosclerotic characteristics, thereby conferring protection against lipoprotein oxidation and the advancement of atherosclerosis (54). HES demonstrates protective properties against atherosclerosis in LDL receptor-deficient (LDLr^−^/^−^) murine models subjected to high-fat diets (HFD). Following a 12-week intervention period, HES significantly mitigated weight gain, enhanced insulin sensitivity, and diminished hyperlipidemic conditions. It effectively inhibited hepatic steatosis, the formation of atherosclerotic plaques, and the development of macrophage foam cells. At the mechanistic level, HES was found to downregulate ACCα and FAS, which are pivotal enzymes involved in fatty acid biosynthesis, while concurrently upregulating ABCG8, ABCA1, and ABCG1, thereby facilitating reverse cholesterol transport. Furthermore, HES restored the activities of antioxidant enzymes and attenuated inflammatory responses. Collectively, these findings suggested that HES alleviates atherosclerosis through a multifaceted approach, encompassing enhancements in metabolic function, lipid homeostasis, antioxidant effects, and anti-inflammatory mechanisms (42).

In ApoE^−^/^−^ murine models, HES enhanced metabolic and physiological well-being, mitigated the formation of atherosclerotic plaques, and attenuated systemic inflammation and oxidative stress (55). Importantly, HES influenced the composition of the gut microbiota, resulting in an increase in beneficial microbial taxa such as Verrucomicrobia and Bacteroidota, while concurrently achieving a significant reduction in fecal branched-chain amino acids (BCAAs), specifically, valine, leucine, and isoleucine, by 27.4, 50.1, and 40.8%, respectively. These results exhibited that HES may alleviate atherosclerosis, at least in part, via the gut microbiota–BCAA–host axis, thereby underscoring its potential as a dietary or therapeutic intervention (55). Varenicline, an agent utilized for smoking cessation, has been demonstrated to facilitate the formation of atherosclerotic plaques in ApoE^−^/^−^ murine models, likely via the enhanced uptake of oxidized low-density lipoprotein (oxLDL) by macrophages through the upregulation of scavenger receptors (CD36, LOX-1) and the downregulation of cholesterol efflux transporters (ABCA1, ABCG1) (56). This investigation illustrated that HES, a flavonoid derived from citrus, significantly inhibited the progression of varenicline-induced plaques in the aorta and conferred protection against the accumulation of oxLDL within macrophages by normalizing the expression levels of these receptors and transporters. These data indicated that HES has the potential to prevent or mitigate cardiovascular risks associated with varenicline administration (56).

While statins are frequently prescribed, they have been associated with hepatotoxicity and myotoxicity, thereby fostering an exploration into botanical substitutes. Research examined the phytochemical constituents and anti-atherosclerotic properties of *Amaranthus viridis* (*A. viridis*) in hypercholesterolemic rabbits (57). Gas Chromatography–Mass Spectrometry/Mass Spectrometry (GC–MS/MS) identified a total of 30 distinct compounds, while Reverse Phase High-Performance Liquid Chromatography (RP-HPLC) successfully detected ascorbic acid, rutin, quercetin, and catechin (57). Administration of *A. viridis* extract resulted in a significant reduction in total cholesterol, LDL-C, and triglycerides, alongside an elevation in HDL-C levels and antioxidant enzyme activities, specifically SOD and GPx, while concurrently inhibiting the formation of aortic plaques and reducing the intima-to-media thickness ratio. These results shown that *A. viridis* and its bioactive phytochemical constituents possess substantial therapeutic potential as natural alternatives in the management of hypercholesterolemia and atherosclerosis (57).

Overall, the contemporary research underscores the promising role of natural compounds in the prevention and mitigation of atherosclerosis through various biological mechanisms. HES demonstrates anti-atherosclerotic properties by modulating cholesterol metabolism, diminishing oxidative stress, inhibiting inflammatory pathways, and regulating the gut microbiome. Furthermore, HES mitigates varenicline-induced atherosclerosis by normalizing macrophage scavenger receptors and cholesterol efflux transporters. In addition, compounds derived from *Vitex rotundifolia* (casticin and luteolin) confer protection to LDL and HDL against oxidative modifications, while the extract from *Amaranthus viridis* enhances lipid profiles, bolsters antioxidant defenses, and decreases aortic plaque formation in hypercholesterolemic models. Collectively, these findings substantiate the therapeutic and preventative promise of flavonoids and plant-derived compounds as natural interventions against atherosclerosis and CVDs.

## 4 The effects of naringin, HES, and HST on cardiac remodeling

Cardiac remodeling, characterized by myocardial hypertrophy, fibrosis, oxidative stress, inflammation, and impaired functionality, represents a fundamental mechanism in the advancement of heart failure and maladaptive cardiovascular responses (58, 59). Naturally occurring flavonoids derived from citrus fruits have surfaced as promising regulators of these biological processes. Naringin has been demonstrated to alleviate structural alterations in the cardiac and aortic tissues precipitated by hypertension (60). HES has exhibited significant effects in models of cardiovascular remodeling. Furthermore, HES has been shown to alleviate cardiac fibrosis in murine subjects exposed to β-adrenergic stimulation by influencing gut microbiota, thereby establishing a connection between microbial alterations and structural cardiac remodeling (61). HST, the aglycone form of HES, has been evaluated in a mouse model of pressure overload (aortic banding).

### 4.1 Studies investigating the cardiac remodeling effects of naringin

Naringin exhibits protective effects against cardiovascular dysfunction and remodeling in hypertensive rats induced by L-NAME (62).

In a longitudinal study spanning five weeks, naringin (20–40 mg/kg) effectively inhibited the elevation of blood pressure, safeguarded left ventricular function (as measured by fractional shortening and ejection fraction), and preserved aortic endothelial function. Furthermore, it inhibited left ventricular and aortic hypertrophy and fibrosis observed in untreated hypertensive rat models. At a mechanistic level, naringin was found to reduce markers associated with the renin-angiotensin system (RAS), oxidative stress, and TNF-α, while concurrently downregulating the AT1R/PKC/NOX2/Raf-1/ERK1/2 signaling pathway within the cardiac tissue. These findings suggested that naringin plays a significant role in alleviating hypertensive cardiac remodeling and dysfunction, indicating its potential utility as a therapeutic agent for the management of hypertension (62) (Table 2). Zhishi Xiebai Guizhi Decoction (ZXGD), a formulation grounded in traditional Chinese medicine, exhibits significant therapeutic promise in the treatment of pulmonary hypertension (PH). In experimental rat models, ZXGD has been observed to diminish pulmonary artery pressure and mitigate pulmonary vascular remodeling. At a mechanistic level, ZXGD has been shown to inhibit the aberrant proliferation of pulmonary artery smooth muscle cells (PASMCs), modulate key factors such as HIF-1α, reactive oxygen species (ROS), and Nrf2 to alleviate conditions of hypoxia and oxidative stress, and regulate pro-inflammatory cytokines to diminish inflammatory responses. Furthermore, ZXGD has been found to enhance lipid metabolism by decreasing levels of decadienyl-L-carnitine and LDL-C, while simultaneously increasing HDL-C and sustaining mitochondrial functionality. Neohesperidin and naringin have been identified as pivotal bioactive constituents that underlie these observed effects. These findings substantiate the potential of ZXGD as a promising candidate for the development of innovative anti-pulmonary hypertension therapies (63).

**Table 2:** Summary of studies investigating the effects of naringin, hesperidin, and hesperetin on cardiac remodeling.

Compound(s)	Experimental model/approach	Intervention duration	Main findings	Proposed mechanisms	Reference
Naringin	L-NAME-induced hypertensive rats (*in vivo*)	4 weeks concurrent with L-NAME administration	Naringin prevented BP elevation, preserved LV function, reduced fibrosis and hypertrophy	Suppression of RAS parameters, oxidative stress, inflammation; restoration of AT1R/PKC/NOX2/Raf-1/ERK1/2 signaling	(62)
Naringin (with neohesperidin)	Pulmonary hypertension rats + PASMCs (*in vivo* and *in vitro*)	3–4 weeks in vivo treatment; 24–48 h PASMC exposure in vitro	ZXGD reduced pulmonary artery pressure, attenuated vascular remodeling, decreased lipid toxicity	Regulation of HIF-1α, ROS, Nrf2; modulation of inflammatory cytokines; reduction of cholesterol/lipid toxicity	(63)
Hesperidin	HFFD-fed Sprague–Dawley rats (*in vivo*)	8–12 weeks concurrent with high-fat/fructose diet	Regulation of HIF-1α, ROS, Nrf2; modulation of inflammatory cytokines; reduction of cholesterol/lipid toxicity	Modulation of AdipoR1, eNOS; suppression of TNF-α and IL-6; antioxidant and anti-inflammatory effects	(41)
Hesperidin	LAD occlusion rats (*in vivo*)	2–4 weeks pretreatment prior to LAD ligation; evaluation up to 24 h–4 weeks post-MI	Pre-treatment with yuzu or hesperidin attenuated cardiac dysfunction, apoptosis, fibrosis	Inhibition of caspase-3, myeloperoxidase, α-SMA, MMP-2; anti-apoptotic and anti-inflammatory effects	(64)
Hesperidin	Aortic banding (AB) mice (*in vivo*)	4–8 weeks post-AB surgery treatment	Hesperetin reduced cardiac hypertrophy, fibrosis, oxidative stress, and myocyte apoptosis	Inhibition of PKCα/βII-AKT, JNK, TGFβ1-Smad signaling pathways	(23)

ABCA1, ATP-binding cassette transporter A1; ACCα, Acetyl-CoA carboxylase alpha; α-SMA, alpha-smooth muscle actin; AKT, protein kinase B; AT1R, angiotensin II receptor type I; ERK1/2, extracellular signal-regulated kinase ½; HFFD, High-fat/high-fructose diet; HIF-1α, Hypoxia-inducible factor 1 alpha; LAD, left anterior descending coronary artery; LV, Left ventricle; MMP-2, matrix metalloproteinase-2; Nrf2, Nuclear factor erythroid 2–related factor 2; NOX2, NADPH oxidase 2; PASMC, Pulmonary artery smooth muscle cell; PKC, Protein kinase C; RAS, Renin-angiotensin system; ROS, reactive oxygen species; TGFβ1, transforming growth factor beta 1; TNF-α, tumor necrosis factor-alpha; ZXGD, Zhishi Xiebai Guizhi Decoction.

### 4.2 Studies investigating the cardiac remodeling effects of HES, and HST

**Figure 3:**
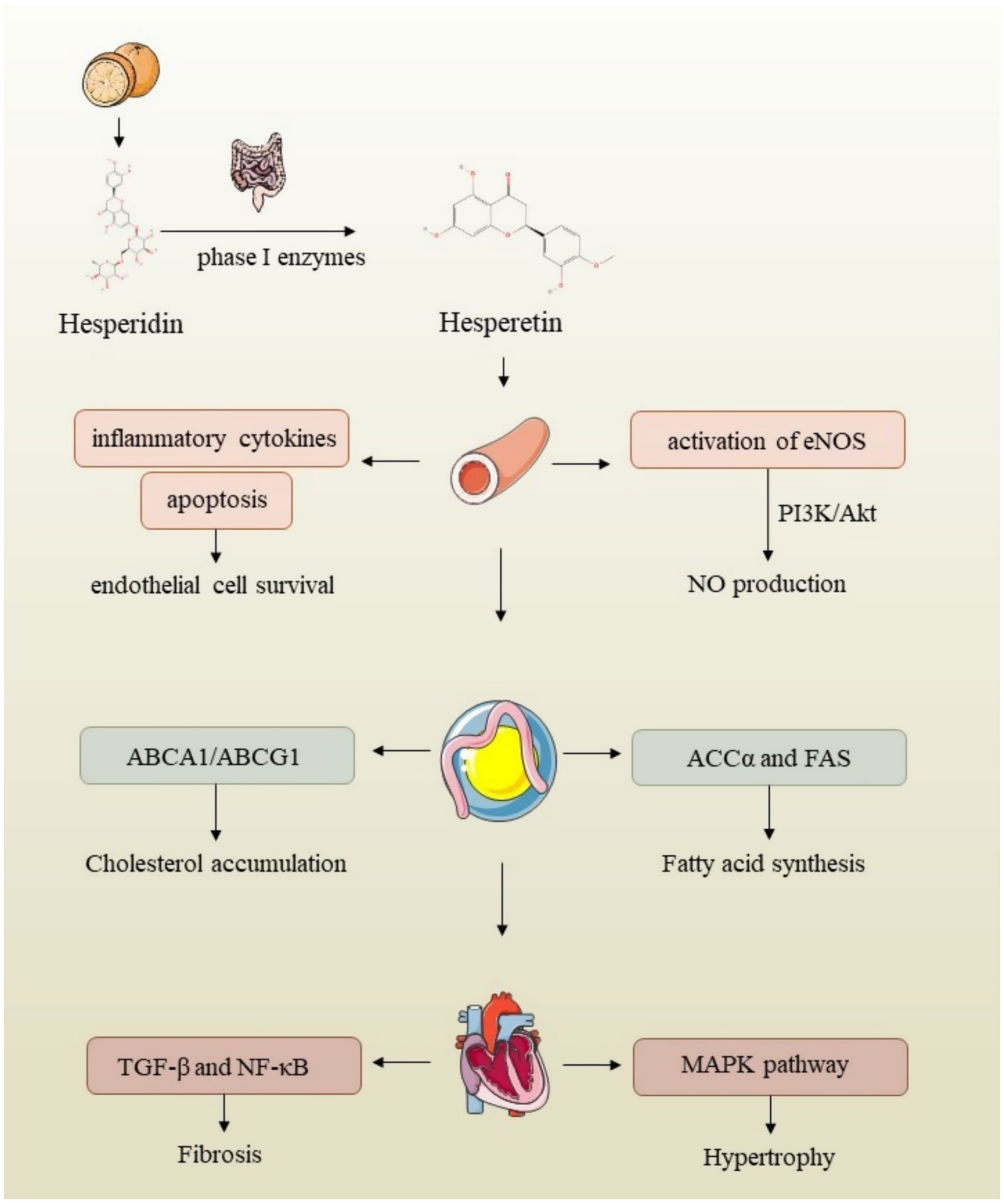
Hesperidin and its aglycone hesperetin protect against cardiac remodeling through endothelial and metabolic regulation. Following intestinal conversion to hesperetin, hesperidin enhances endothelial function (↑eNOS, ↑NO), inhibits inflammatory cytokines (↓TNF-α, ↓IL-6), and improves lipid handling by stimulating cholesterol efflux transporters (ABCA1/ABCG1) and suppressing fatty-acid synthesis (ACCα, FAS). These actions, mediated via Sirt1/Nrf2/HO-1 and PI3K/Akt/mTOR pathways, collectively reduce oxidative stress, fibrosis, and hypertrophy.

HES demonstrates protective properties against vascular dysfunction and structural remodeling in rats subjected to a high-fat/high-fructose dietary regimen (HFFD) (41). Administration of HES (30 mg/kg/day) over a period of four weeks mitigated metabolic irregularities, enhanced endothelial functionality, and diminished vascular remodeling (Figure 3). From a mechanistic perspective, HES reduced oxidative stress (as indicated by plasma malondialdehyde (MDA) and aortic superoxide levels) and inflammation (notably TNF-α and IL-6), while simultaneously increasing plasma adiponectin, nitric oxide metabolites, and reinstating the expression of AdipoR1 and eNOS in the aorta. These results exhibited that HES mitigates dietary-induced vascular dysfunction and remodeling through mechanisms that are anti-oxidative, anti-inflammatory, and protective of endothelial cells (41). Yuzu, a citrus fruit abundant in HES, demonstrates protective effects against left ventricular (LV) remodeling subsequent to myocardial infarction (MI).

In a rat model characterized by permanent left anterior descending (LAD) coronary artery occlusion, pre-treatment with yuzu or HES for a duration of one week mitigated cardiac dysfunction, myocyte apoptosis, and inflammatory responses. Both yuzu and HES exhibited inhibitory effects on caspase-3 activity, myeloperoxidase expression, α-smooth muscle actin expression, and MMP-2 activity, thereby precluding structural remodeling of the myocardium. These results shown that yuzu and HES possess the capability to avert MI-induced LV remodeling and subsequent ventricular dysfunction (64). HST exhibits protective effects against cardiac remodeling that is instigated by pressure overload. In a murine model subjected to aortic banding (AB), the administration of HST via oral route significantly mitigated cardiac hypertrophy, fibrosis, oxidative stress, and myocyte apoptosis, thereby enhancing cardiac function as evaluated through echocardiography, hemodynamics, histopathological analysis, and molecular biomarkers. From a mechanistic standpoint, HST effectively inhibited the PKCα/βII-AKT, JNK, and TGFβ1-Smad signaling cascades, which are intricately involved in the processes of hypertrophy and fibrosis. These findings demonstrated that HST possesses the potential to function as a therapeutic agent for the management of cardiac remodeling and heart failure (23). Collectively, the flavonoids naringin, HES, and HST demonstrate protective properties against cardiac remodeling through the inhibition of hypertrophy, fibrosis, oxidative stress, and inflammation, as well as the modulation of critical signaling pathways and, in certain instances, the gut-cardiac axis. Notably, HES and HST exhibit particularly robust effects in models of structural remodeling, whereas naringin reveals encouraging outcomes in models of hypertrophy and hypertension. These observations provide a compelling basis for the advancement of research into citrus flavonoids as potential adjunctive therapies for pathological cardiac remodeling and heart failure.

## 5 The effects of naringin, HES, and HST on myocardial ischemia

Naringin, HES, and HST, which are three notable citrus-derived flavonoids, have been shown to possess considerable cardioprotective properties in the context of myocardial ischemia as well as ischemia/reperfusion (I/R) injury. Naringin has been evidenced to safeguard cardiac architecture and functionality by mitigating oxidative stress, inflammation, apoptosis, and ferroptosis in both *in vivo* and *in vitro* experimental models of myocardial I/R injury. At the mechanistic level, it influences signaling pathways including PI3K/Akt, IRF3/SLC7A11/Gpx4, and cGAS-STING, thereby enhancing mitochondrial efficacy and diminishing myocardial cell mortality (65, 66). HES similarly manifests cardioprotective properties through the attenuation of oxidative stress, inflammatory cytokine production, and apoptosis, while also modulating autophagy via the activation of the PI3K/Akt/mTOR signaling cascade, which collectively improve cardiac outcomes following myocardial ischemia (67). HST, which represents the aglycone derivative of HES, has been documented to enhance myocardial viability, diminish infarct size, suppress inflammatory responses, and mitigate cardiac remodeling through the modulation of oxidative stress, mitochondrial function, Ca^2+^ influx, and NF-κB signaling pathways (37, 66, 68). Collectively, these flavonoids demonstrate multi-targeted effects in the prophylaxis and treatment of myocardial ischemia, emphasizing their prospective utility as dietary or pharmacological interventions for ischemic heart disease. Their antioxidative, anti-inflammatory, anti-apoptotic, and lipid-regulating attributes render them promising candidates for alleviating ischemia-induced cardiac injury and remodeling, thereby providing a foundational basis for forthcoming translational and clinical investigations.

### 5.1 Effects of naringin on myocardial ischemia: evidence from experimental studies

Naringin demonstrates cardioprotective properties in the context of myocardial ischemia–reperfusion injury (MI/RI) by specifically targeting cardiac microvascular endothelial cells (CMECs), which are essential for the preservation of myocardial perfusion and functionality. Empirical investigations conducted in rat models of MI/RI and CMEC hypoxia-reoxygenation (H/R) assays indicate that naringin sustains microvascular integrity through the inhibition of ferroptosis, a regulated mechanism of cell death associated with ischemic injury. From a mechanistic standpoint, naringin activates the IRF3/SLC7A11/Gpx4 signaling pathway, thereby enhancing antioxidant defenses and facilitating the mitochondrial translocation of NDUFS1, which subsequently improves mitochondrial functionality. Collectively, these ramifications mitigate CMEC ferroptosis, restore mitochondrial performance, and uphold cardiac microvascular functionality, underscoring naringin’s potential as a therapeutic agent for safeguarding the heart against MI/RI (65) (Table 3). In addition, naringin confers protection against myocardial ischemia–reperfusion injury (MI/RI) through the modulation of mitochondrial functionality and the process of ferroptosis. In studies involving H9c2 cardiomyocytes as well as rat models of MI/RI, the application of naringin at a concentration of 480 μM was found to be non-toxic and effective in preserving cellular viability, concurrently diminishing oxidative stress and damage instigated by ferroptosis. From a mechanistic perspective, naringin exerts its effects by inhibiting the cGAS-STING signaling pathway, a mechanism that underpins its cardioprotective properties, as corroborated by *in vivo* investigations. These data underscore naringin’s potential as a therapeutic agent for alleviating MI/RI through its regulatory influence on mitochondrial dysfunction, ferroptosis, and inflammatory signaling pathways (66).

**Table 3:** Summary of studies investigating the effects of naringin, hesperidin, and hesperetin on myocardial ischemia.

Flavonoid	Model/species	Dose/duration	Mechanism/pathway	Key findings/outcomes	Reference
Naringin	Rat MI/RI; CMECs H/R	Rats: LAD ligation 45 min + 6 h reperfusion; *In vitro*: 12 h hypoxia + 24 h reoxygenation	IRF3/SLC7A11/Gpx4; mitochondrial NDUFS1 translocation	Enhanced microvascular function; inhibited CMEC ferroptosis; improved mitochondrial function	(65)
Naringin	H9c2 cardiomyocytes H/R; Rat MI/RI	480 μM Nar *in vitro*	cGAS-STING pathway	Reduced myocardial injury; improved mitochondrial function; inhibited ferroptosis	(66)
Naringin	Rat global ischemia/reperfusion	Pretreatment 25 mg/kg for 7 days	KATP channels; antioxidant; anti-apoptotic	Restored cardiac function; reduced arrhythmia, ROS, lipid peroxidation; downregulated Bax/Bcl-2	(69)
Naringin	Rat MI/RI	ZQAE 50–100 mg/kg, Naringin 5 mg/kg, 5-day pretreatment	SIRT1/HMGB1; anti-apoptotic, anti-inflammatory, antioxidant	Reduced infarct size; decreased myocardial enzymes; suppressed apoptosis, inflammation, oxidative stress	(70)
Naringin	AC16 cells OGD/R; Rat MI/RI	Naringin pretreatment; miR-126 agomir *in vivo*	miR-126/GSK-3β/β-catenin	Decreased cardiomyocyte apoptosis & cytokine release; upregulated miR-126; inhibited GSK-3β; activated β-catenin	(71)
Naringin	Rat MI/RI	100 mg/kg Naringin	PI3K/Akt; inhibition of apoptosis, oxidative stress, autophagy	Reduced myocardial enzymes, apoptosis, oxidative stress, infarct size; improved LV function; modulated LC3BII/LC3BI and Beclin-1	(24)
Naringin	Rat MI/RI	20–80 mg/kg/day for 14 days	Hsp27/Hsp70; p-Akt/p-eNOS/p-ERK; anti-apoptotic; anti-inflammatory	Improved cardiac function; reduced infarct size; increased NO bioavailability; regulated apoptotic and inflammatory markers	(72)
Hesperetin	ISO-induced myocardial ischemia in mice	25–50 mg/kg/day for 7 days	Sirt1/Nrf2 pathway; antioxidant, anti-inflammatory, anti-apoptotic	Reduced oxidative stress, inflammation, apoptosis; improved ECG and cardiac histology; modulated Bax/Bcl-2, caspase-3, Sirt1/Nrf2 proteins	(73)
Neohesperidin	Rat MI/RI	Not specified	JNK & NF-κB p65 inhibition	Suppressed myocardial injury, apoptosis, oxidative stress, inflammatory cytokines; restored immunological balance	(74)
Hesperidin	Rat MI/RI	Pretreatment 3 days; dose not specified	PI3K/Akt/mTOR; inhibition of excessive autophagy	Decreased infarct size, myocardial damage, CK-MB, cTnI; downregulated LC3II and Beclin1; upregulated p-Akt, p-PI3K, p-mTOR	(67)
Hesperidin	Rat MI/RI	200 mg/kg, 3-day pretreatment	PI3K/Akt; HMGB1 inhibition; anti-apoptotic, anti-inflammatory, antioxidant	Reduced infarct size, myocardial enzymes, apoptosis, inflammation, oxidative stress; increased p-Akt; inhibited HMGB1	(75)
Hesperidin	Rat; isoproterenol-induced MI	100–400 mg/kg; optimal 200 mg/kg	Anti-lipid peroxidation; antioxidant	Normalized lipid peroxidation; restored enzymatic and non-enzymatic antioxidants; reduced myocardial damage	(76)
Hesperidin	Rat MI/RI	Not specified	Anti-inflammatory; antioxidant; anti-apoptotic	Decreased infarct size, arrhythmias, apoptosis; increased antioxidant activity; reduced inflammation	(77)
Hesperidin	H9c2 cardiomyocytes I/H	CoCl2 22 h; HSP 4 h treatment	Oxidative stress inhibition; apoptosis; Ca2 + homeostasis; LTCCs	Increased cell viability & MMP; reduced ROS, apoptosis, [Ca2+]i; improved myocardial contraction & Ca2 + transients	(78)

MI, myocardial infarction; MI/RI, myocardial ischemia–reperfusion injury; Nar, naringin; HSP, hesperetin; Hsd, hesperidin; NEO, neohesperidin; CMECs, cardiac microvascular endothelial cells; H/R, hypoxia/reoxygenation; LAD, left anterior descending artery; ECG, electrocardiogram; ROS, reactive oxygen species; SOD, superoxide dismutase; Bax, Bcl-2 associated X protein; Bcl-2, B-cell lymphoma 2; NAC, N-acetylcysteine; GLI, glibenclamide; ZQAE, aqueous extract of citrus Wilsonii Tanaka; SIRT1, Sirtuin 1; HMGB1, high mobility group box 1 protein; OGD/R, oxygen–glucose deprivation/reoxygenation; GSK-3β, glycogen synthase kinase 3 beta; PI3K, phosphatidylinositol 3-kinase; Akt, protein kinase B; LC3B, microtubule-associated protein 1 light chain 3B; Beclin-1, autophagy-related protein beclin-1; Hsp27/Hsp70, heat shock protein 27/70; p-Akt /p-eNOS/p-ERK, phosphorylated Akt/endothelial nitric oxide synthase/extracellular signal-Regulated kinase; ISO, isoproterenol; Nrf2, nuclear factor erythroid 2–related factor 2; TNF-α, tumor necrosis factor alpha; IL-6/IL-8/IL-23, interleukin 6/8/23; NF-κB, nuclear factor kappa B; JNK – c, Jun N-terminal kinase; LTCCs, L-type calcium channels; MMP, mitochondrial membrane potential; [Ca^2+^]I, intracellular calcium concentration; CK-MB, creatine kinase-MB; cTnI/cTnT, cardiac troponin I/T; PI3K/Akt/mTOR, phosphatidylinositol 3-kinase/protein kinase B/mammalian target of rapamycin.

In experimental studies utilizing rat models, pretreatment with NRG effectively restored cardiac function and corrected electrocardiographic alterations, diminished the incidence of arrhythmias, reduced infarct size, and decreased lactate dehydrogenase release, while concurrently enhancing the activities of antioxidant enzymes such as SOD, CAT, and citrate synthase. On a mechanistic level, NRG attenuated the generation of ROS, mitigated lipid peroxidation, and inhibited apoptosis through the downregulation of Bax and the Bax/Bcl-2 ratio, in addition to activating K-ATP channels, with glibenclamide partially obstructing these protective effects. Histopathological examinations corroborated the reduction of cardiomyocyte edema and hypertrophy. Collectively, these findings exhibited that NRG confers protection to the heart from I/R injury via antioxidant, antiapoptotic, and K-ATP channel-mediated pathways (69). In rodent experimental models, pre-administration of naringin or the aqueous extract derived from fruit (ZQAE) yielded significant improvements in histopathological parameters, attenuated the release of myocardial enzymes (cTnI, CK-MB, CK, LDH), and diminished the extent of infarct size. From a mechanistic standpoint, naringin was found to inhibit apoptosis (by downregulating cleaved-caspase3 and the Bax/Bcl2 ratio), suppress inflammatory responses (as indicated by levels of IL-6, IL-23, TNF-α), and mitigate oxidative stress (as evidenced by measurements of MDA and SOD). The cardioprotective effects observed were linked to the activation of SIRT1 and the inhibition of HMGB1, thereby suggesting that naringin alleviates myocardial ischemia/reperfusion injury through mechanisms that involve anti-apoptotic, anti-inflammatory, and antioxidant pathways. Its therapeutic efficacy was found to be comparable to that of the standard pharmacological agent diltiazem when administered in a short-term pretreatment regimen (70).

In addition, naringin exhibits considerable cardioprotective properties against myocardial ischemia–reperfusion (I/R) injury through mechanisms that are both anti-apoptotic and anti-inflammatory in nature. In human AC16 cardiomyocytes subjected to oxygen–glucose deprivation and subsequent recovery (OGD/R), pretreatment with naringin resulted in a significant reduction in apoptosis and a decrease in the secretion of pro-inflammatory cytokines, including IL-6, IL-8, and TNF-α. From a mechanistic perspective, naringin was found to upregulate microRNA-126 (miR-126), which directly targets and inhibits GSK-3β, thereby facilitating the activation of the β-catenin signaling pathway, which promotes cellular survival and mitigates injury. *In vivo* investigations utilizing rat models of myocardial I/R corroborated that both naringin pretreatment and miR-126 agomir markedly reduced myocardial injury, enhanced histological outcomes, and preserved cardiac function. These observations shown that the cardioprotective effect of naringin involves the modulation of the miR-126/GSK-3β/β-catenin axis, thereby presenting a potential therapeutic strategy for acute myocardial infarction and myocardial I/R injury (71). In rodent experimental models, the administration of (NRG) at a carefully calibrated dosage of 100 mg/kg resulted in a statistically significant reduction in the release of myocardial enzymes, a decrease in infarct size, and a mitigation of apoptosis and inflammation, concurrently enhancing cardiac function as evidenced by elevated left ventricular ejection fraction and fractional shortening measurements. From a mechanistic perspective, NRG elicited activation of the phosphoinositide 3-kinase (PI3K)/Akt signaling pathway, which catalyzed the phosphorylation of Akt and inhibited autophagy markers, notably including beclin-1 and the LC3B II/I ratio. The cardioprotective outcomes were partially negated by the application of the PI3K/Akt inhibitor LY294002, thereby substantiating the role of this signaling pathway. Collectively, these observations indicated that NRG confers cardioprotection against I/R injury through the orchestration of Akt-mediated signaling, the reduction of autophagy, oxidative stress, apoptosis, and inflammation, thereby underscoring its potential therapeutic applications in the context of ischemic heart diseases (24). In experimental rat models, the oral administration of naringin (20–80 mg/kg/day) for a duration of 14 days preceding IR significantly enhanced cardiac function, which encompassed mean arterial pressure, heart rate, inotropic (+LVdP/dt max) and lusitropic (-LVdP/dt max) conditions, as well as diminished left ventricular end-diastolic pressure. Naringin effectively reduced infarct size and preserved the ultrastructural integrity of myocardial tissue. Mechanistically, it augmented the bioavailability of nitric oxide, upregulated the expression of Hsp27, Hsp70, and β-catenin, and activated signaling pathways involving p-eNOS, p-Akt, and p-ERK, while concurrently inhibiting the TNF-α/IKK-β/NF-κB and JNK cascades. Furthermore, naringin modulated apoptotic processes by elevating Bcl-2 levels and decreasing the expression of Bax and caspase-3, reduced TUNEL positivity, normalized cardiac injury biomarkers (LDH, CK-MB), reinstated antioxidant defenses, and mitigated lipid peroxidation. These findings elucidated that naringin ameliorates myocardial structural and functional integrity through the modulation of oxidative stress, inflammatory signaling, and apoptotic pathways, thereby underscoring its therapeutic potential in the context of myocardial ischemia–reperfusion injury (72).

### 5.2 Effects of HES and HST on myocardial ischemia: evidence from experimental studies

HES exhibits notable cardioprotective properties against myocardial ischemia precipitated by isoproterenol (ISO) in murine models. In controlled experimental settings, administration of HSP at both low (25 mg/kg/day) and high (50 mg/kg/day) dosages resulted in significant enhancements in electrocardiographic metrics, a reduction in heart rate, and amelioration of histopathological changes within cardiac tissues. Treatment with HSP diminished oxidative stress, as evidenced by a reduction in MDA levels and an elevation in the activities of SOD, CAT, and glutathione. Furthermore, it effectively attenuated inflammatory responses, as indicated by decreased serum concentrations of IL-6 and TNF-α. In addition, HSP inhibited myocardial apoptosis by modulating the expressions of Bax, Bcl-2, and caspase-3. The underlying mechanisms of these protective effects were orchestrated through the activation of the Sirt1/Nrf2 signaling pathway, which in turn enhanced downstream antioxidant responses (NQO-1, HO-1) and promoted cellular viability. Collectively, HSP confers cardioprotection through the reduction of oxidative stress, inflammation, and apoptosis, thereby underscoring its potential utility as a therapeutic agent for ischemic heart disease (73). Neohesperidin (NEO), a flavonoid derived from citrus, demonstrates cardioprotective properties against myocardial I/R injury through its antioxidant, anti-inflammatory, and anti-apoptotic mechanisms. In experimental rat models of I/R, NEO significantly diminished serum concentrations of inflammatory cytokines, markers indicative of myocardial injury, and indicators of oxidative stress, whilst simultaneously enhancing the endogenous antioxidant capacity. Additionally, NEO suppressed cardiomyocyte apoptosis and positively influenced indices of immune organs, such as the spleen and thymus, as well as enhancing phagocytic activity. Mechanistically, these protective effects were facilitated through the attenuation of the JNK and NF-κB signaling pathways, as the advantages conferred by NEO were negated by the activation of JNK or NF-κB. By alleviating oxidative stress, inflammation, apoptosis, and immunological dysregulation, NEO exemplifies a multi-faceted mechanism of myocardial protection, thereby underscoring its prospective development as a therapeutic intervention for myocardial I/R injury (74).

In a rat model of I/R, the administration of HES prior to the event significantly mitigated the size of myocardial infarction, enhanced the histological integrity of cardiac tissue, and resulted in a reduction of serum concentrations of CK-MB and cardiac troponin I, thereby indicating a diminished level of myocardial injury. From a mechanistic standpoint, HES inhibited the exacerbation of autophagy through the downregulation of LC3II and Beclin1, while simultaneously promoting the activation of the PI3K/Akt/mTOR signaling cascade, as demonstrated by the increased phosphorylation of PI3K, Akt, and mTOR. The cardioprotective effects were effectively nullified upon the inhibition of PI3K by LY294002, thereby affirming the pivotal role of the PI3K/Akt/mTOR pathway in the regulatory effects of HES on autophagy. These data exhibited that HES diminishes myocardial I/R injury through the attenuation of excessive autophagy via the activation of the PI3K/Akt/mTOR pathway, thereby underscoring its potential as a viable therapeutic agent for the treatment of ischemic heart diseases (67). In a rat experimental model, a short-term (3-day) administration of 200 mg/kg HES was established as the most effective dosage, significantly diminishing infarct size, myocardial enzyme concentrations, apoptosis, inflammation, and oxidative stress. Mechanistically, HES was found to downregulate the expression of HMGB1 while concurrently activating the PI3K/Akt signaling pathway. The inhibition of PI3K using LY294002 partially counteracted these effects, thereby illustrating that the cardioprotective properties of HES are facilitated through the PI3K/Akt-dependent attenuation of HMGB1, which subsequently alleviates apoptosis, inflammatory responses, and oxidative damage within myocardial tissue. These findings emphasize the potential of HES as a short-term prophylactic strategy against ischemia/reperfusion injury and underscore the critical significance of the PI3K/Akt-HMGB1 pathway in the mechanism underlying its cardioprotective effects (75).

HES demonstrates considerable cardioprotective attributes in the context of ISO-induced myocardial ischemia in rodent models. The administration of ISO resulted in significant myocardial injury, as evidenced by elevated cardiac biomarkers (AST, ALT, LDH, CK, CK-MB, cTnT, cTnI), heightened lipid peroxidation (TBARS, lipid hydroperoxides, conjugated dienes), and diminished levels of both non-enzymatic antioxidants (vitamin C, vitamin E, GSH) and enzymatic antioxidants (SOD, CAT, GPx, GST, GR). Intervention with HES, notably at a dosage of 200 mg/kg, led to the normalization of cardiac biomarkers, a reduction in oxidative stress, a restoration of antioxidant defenses, and an inhibition of lipid peroxidation, thereby illustrating a thorough protective mechanism. These observations exhibited that the cardioprotective actions of HES in myocardial ischemia are predominantly facilitated through its antioxidant and anti-lipid peroxidative characteristics, underscoring its potential as a therapeutic agent to alleviate ischemia-induced myocardial damage (76). HES administration significantly reinstated antioxidant capacity and tissue nitrite concentrations, whilst concurrently diminishing inflammation, myocardial apoptosis, and the occurrence of arrhythmic events. These outcomes imply that HES alleviates ischemia/reperfusion-induced cardiac arrhythmias predominantly through the reduction of oxidative stress and the inhibition of inflammatory responses. By safeguarding myocardial functionality and constraining infarct-associated damage, HES presents itself as a promising therapeutic candidate for the prophylaxis of ischemia/reperfusion-related arrhythmias and acute cardiac events (77). In H9c2 cardiomyocytes exposed to CoCl₂-induced hypoxic conditions, treatment with HSP markedly improved cell viability and mitochondrial membrane potential, whilst concurrently diminishing oxidative stress, apoptosis, and intracellular calcium [(Ca^2+^)ᵢ] overload. Furthermore, HSP exhibited a concentration-dependent attenuation of L-type Ca^2+^ currents (I_Ca-L), myocardial contraction, and Ca^2+^ transients, implying its involvement in the modulation of calcium handling mechanisms. Collectively, these effects serve to alleviate cardiac dysfunction and cellular damage precipitated by ischemia/hypoxia. The investigation underscores that the cardioprotective mechanism of HSP encompasses the restoration of oxidative homeostasis, inhibition of apoptotic pathways, enhancement of mitochondrial functionality, and suppression of calcium influx through L-type calcium channels. These findings advocate for additional research into HSP as a prospective therapeutic agent or LTCC inhibitor for myocardial injury associated with ischemic and hypoxic conditions (78). In conclusion, naringin, HES, and HST manifest significant cardioprotective effects against myocardial ischemia by addressing oxidative stress, inflammation, apoptosis, ferroptosis, and mitochondrial dysfunction. Their multi-targeted mechanisms not only attenuate myocardial injury and infarct size but also enhance cardiac function and avert adverse remodeling, thereby underscoring their potential as natural therapeutic agents for ischemic heart disease.

## 6 The effects of naringin, HES, and HST on myocardial infarction

Naringin, HES, and HST demonstrate considerable cardioprotective properties against MI. Naringin has been evidenced to enhance mitochondrial functionality, mitigate oxidative stress, and diminish apoptosis in ISO-induced MI experimental models, thereby safeguarding cardiac architecture and performance (71, 79). HES manifests anti-inflammatory and antioxidant properties, lowers myocardial enzyme discharge, and restrains excessive autophagy via the PI3K/Akt/mTOR signaling pathway, ultimately leading to a reduction in infarct size and cardiac remodeling (75, 76). HST likewise alleviates post-MI cardiac fibrosis and inflammation through the modulation of the NF-κB signaling cascade and the regulation of oxidative stress markers (80). Collectively, these flavonoids possess the capacity to diminish myocardial damage, augment cardiac functionality, and offer prospective therapeutic advantages for individuals predisposed to MI.

### 6.1 Effects of naringin on myocardial infarction: evidence from experimental studies

Naringin demonstrates a cardioprotective effect that may prevent ISO-induced MI in male Wistar rats. The administration of ISO (85 mg/kg, 2 doses) resulted in considerable mitochondrial oxidative stress, as evidenced by elevated levels of lipid peroxides coupled with diminished activities of mitochondrial antioxidants, including SOD, CAT, GPx, glutathione-S-transferase, and reduced glutathione. Moreover, ISO perturbed mitochondrial lipid equilibrium, leading to an increase in cholesterol, triglycerides, and free fatty acids, while concurrently decreasing phospholipid levels. Oral pretreatment with naringin (10–40 mg/kg/day for 56 days) markedly decreased mitochondrial lipid peroxidation, reinstated antioxidant defenses, and normalized mitochondrial lipid profiles. These findings demonstrated that naringin effectively safeguards mitochondrial integrity from oxidative damage and lipid dysregulation in ISO-induced myocardial injury, underscoring its potential role as a therapeutic agent for myocardial infarction through mechanisms of mitochondrial stabilization and antioxidative activity (79) (Table 4). The administration of ISO at a dosage of 85 mg/kg, administered in two separate doses, resulted in significant myocardial damage, as indicated by elevated serum concentrations of cardiac troponin T (cTnT), lactate dehydrogenase isoenzymes (LDH1 and LDH2), and various cardiac enzyme markers such as creatine kinase (CK), CK-MB, LDH, aspartate aminotransferase (AST), and alanine aminotransferase (ALT), alongside diminished enzymatic activities within the heart and electrocardiogram (ECG) irregularities. Additionally, ISO treatment led to the disruption of lysosomal enzyme activities, which in turn elevated serum and cardiac levels of beta-glucuronidase, beta-N-acetyl glucosaminidase, beta-galactosidase, cathepsin B, and cathepsin D, while concurrently reducing the lysosomal cardiac activities of beta-glucuronidase and cathepsin D. The administration of naringin as an oral pretreatment (doses ranging from 10 to 40 mg/kg/day over a period of 56 days) resulted in the normalization of cardiac biomarkers, the restoration of typical ECG patterns, and the rectification of lysosomal enzyme imbalances. These observations shown that naringin exerts a protective effect against ISO-induced myocardial injury by stabilizing cardiac enzyme levels (81). ISO administration (85 mg/kg, 2 doses) elicited a pronounced dyslipidemic response, characterized by elevated serum and cardiac concentrations of total cholesterol, esterified cholesterol, free cholesterol, triglycerides (TG), and free fatty acids (FFA), concomitant with diminished levels of cardiac phospholipids (PL). Furthermore, ISO perturbed lipid metabolic pathways by modifying serum lipoprotein concentrations and enzymatic activities, notably including 3-hydroxy-3-methylglutaryl-Coenzyme A reductase in hepatic and cardiac tissues, lecithin cholesterol acyltransferase, and lipoprotein lipase in plasma. Preceding oral administration of naringin (10–40 mg/kg/day for a duration of 56 days) effectively reinstated serum and cardiac lipid homeostasis, replenished cardiac phospholipid concentrations, and rectified irregularities in lipid metabolic enzymes. These observations exhibited that naringin may mitigate ISO-induced myocardial lipid dysregulation and could play a significant role in its overarching cardioprotective properties within myocardial infarction models (82).

**Table 4:** Summary of studies investigating the effects of naringin, hesperidin, and hesperetin on myocardial infarction.

Compound	Model/species	Dose and duration	Main findings/Mechanism	Reference
Naringin	Male Wistar rats, ISO-induced MI	10, 20, 40 mg/kg, oral, 56 days	ISO increased mitochondrial lipid peroxides, decreased antioxidants and altered lipid profile; Naringin prevented these changes and restored mitochondrial function.	(79)
Naringin	Male wistar rats, ISO-induced MI	10, 20, 40 mg/kg, oral, 56 days	ISO increased cTnT, CK-MB, LDH, AST, ALT, altered ECG and lysosomal enzymes; Naringin pretreatment normalized cardiac markers, ECG patterns, and lysosomal enzyme activity, showing cardioprotection.	(81)
Naringin	Male wistar rats, ISO-induced MI	10, 20, 40 mg/kg, oral, 56 days	ISO increased serum/heart cholesterol, TG, FFA, decreased phospholipids; Naringin reduced lipid abnormalities and improved lipoproteins and lipid metabolic enzyme activity.	(82)
Hesperidin	Mouse model, AMI	Not specified	Hesperidin reduced infarct size, HW/BW ratio, CK-MB; decreased TNF-α, IL-1β, IL-6, MCP-1, ICAM-1, MDA, caspases; regulated p53, Bax/Bcl-2, and increased PPARγ expression; anti-inflammatory and antioxidant effects.	(83)
Hesperidin	Clinical study, MI patients	600 mg/day, 4 weeks	Hesperidin decreased E-selectin, increased adiponectin and HDL-C; other inflammatory markers improved but not statistically significant; supports cardiovascular benefits of dietary flavonoids.	(84)

AMI, acute myocardial infarction; CK, creatine kinase; CK-MB, creatine kinase-MB isoenzyme; ECG, electrocardiogram; FFA, free fatty acids; HDL-C, high-density lipoprotein cholesterol; HW/BW, heart weight to body weight ratio; ISO, isoproterenol; LDH, lactate dehydrogenase; MDA, malondialdehyde; MI, myocardial infarction; PPARγ, peroxisome proliferator activated receptor gamma; SOD, superoxide dismutase; TG, triglycerides; CAT, catalase; TNF-α, tumor necrosis factor alpha; IL, interleukin; ICAM-1, intercellular adhesion molecule 1.

### 6.2 Effects of HES and HST on myocardial ischemia: evidence from experimental studies

**Figure 4:**
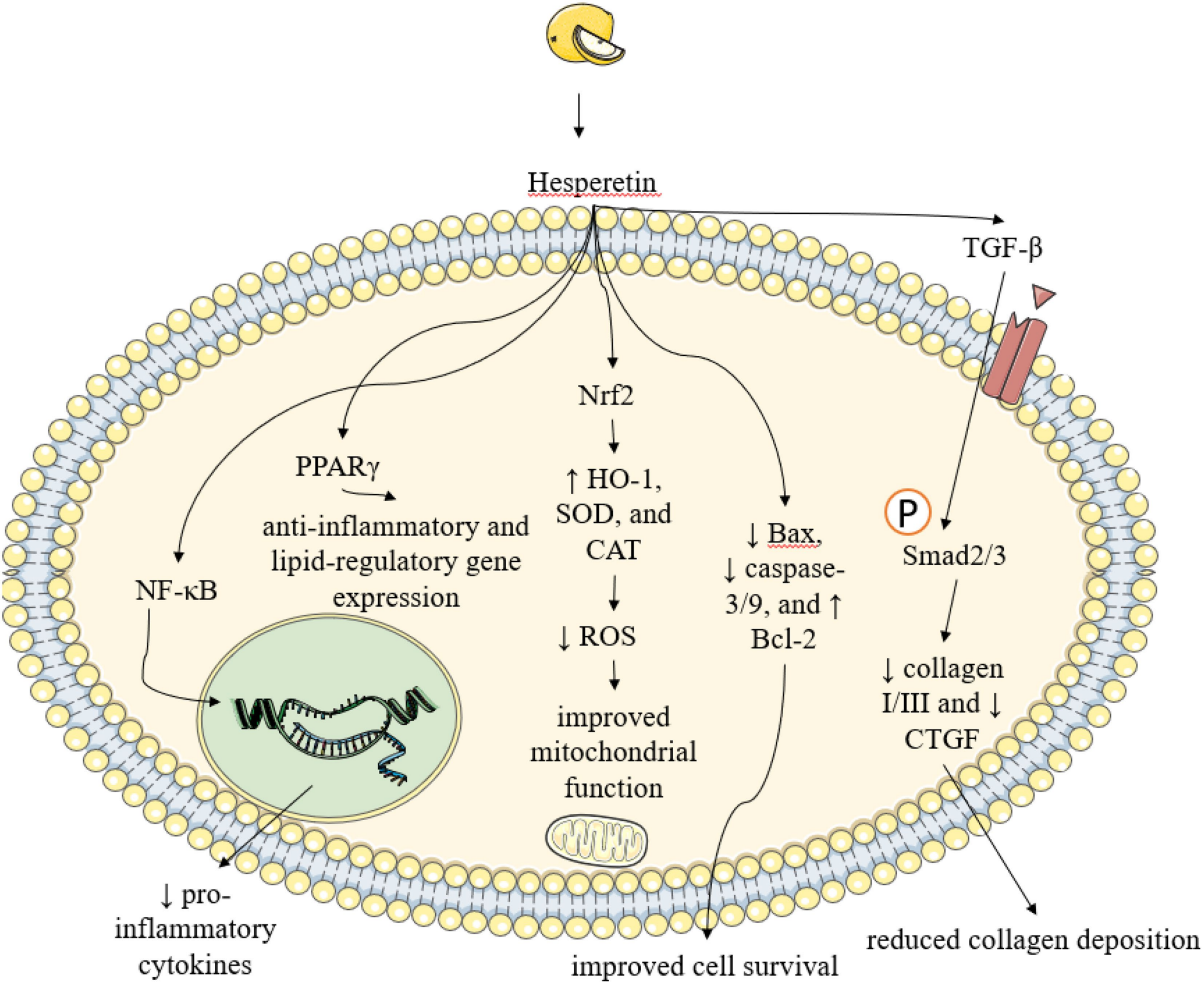
Hesperetin attenuates post-infarction inflammation, fibrosis, and apoptosis through modulation of key signaling pathways. Hesperetin suppresses NF-κB and TGF-β/Smad activation while stimulating Nrf2- and PPARγ-dependent antioxidant and metabolic signaling. These coordinated effects lower ROS levels, inhibit pro-inflammatory and pro-fibrotic gene expression (TNF-α, IL-1β, collagen I/III, CTGF), and restore cellular homeostasis by regulating Bax/Bcl-2 and caspase activity, thereby preserving cardiomyocyte viability.

HST demonstrates notable antioxidant and anti-inflammatory characteristics and possesses prophylactic effects against acute myocardial infarction (AMI) (Figure 4). In a murine model of AMI, HST markedly diminished myocardial infarct size, the heart weight to body weight ratio, and the activity of creatine kinase MB. It attenuated inflammatory responses by downregulating TNF-α, IL-1β, IL-6, MCP-1, and ICAM-1, while simultaneously enhancing oxidative equilibrium through the modulation of MDA, CAT, and SOD levels. HST additionally curtailed apoptosis, as demonstrated by a reduction in caspase-3/9 activity and the downregulation of p53 and Bax/Bcl-2 expression. Moreover, it stimulated the expression of PPARγ, thereby contributing to cardioprotection. Collectively, HST alleviates AMI-induced cardiac injury via anti-inflammatory, antioxidant, and anti-apoptotic pathways, underscoring its therapeutic potential in the prevention of myocardial damage and the preservation of cardiac functionality (83). In a meticulously designed randomized, double-blind clinical trial encompassing 75 MI patients, the administration of 600 mg of HES on a daily basis over a four-week period resulted in a statistically significant decrement in serum E-selectin levels, alongside an elevation in adiponectin and HDL-C concentrations. Furthermore, enhancements in various other inflammatory biomarkers, such as IL-6, high-sensitivity C-reactive protein (hs-CRP), and leptin, in addition to alterations in lipid profiles, were recorded; however, the differences when compared to the placebo group did not achieve statistical significance. These observations elucidate that HES possesses the capacity to modulate pivotal inflammatory mediators and adipocytokines, thereby contributing to cardiovascular protection. The outcomes of this investigation substantiate the prospective role of dietary flavonoids in mitigating post-MI inflammatory responses, optimizing lipid metabolism, and promoting overall cardiac health, thereby underscoring HES as a potentially valuable adjunctive therapeutic agent in the management of myocardial infarction (84).

An investigation assessed the cardioprotective properties of HES in the context of ISO-induced MI in both normoglycemic and Streptozotocin-Nicotinamide (STZ-NIC) induced diabetic rat models. HES (100 mg/kg, p.o.) was administered over a period of 28 days preceding the induction of myocardial infarction. The diabetic rat cohort exhibited pronounced hyperglycemia, elevated levels of HbA1c, TC, TG, and LDL-C, alongside an increase in heart tissue cholesterol ester synthetase (CES), a decrease in lecithin cholesterol acyltransferase (LCAT) and lipoprotein lipase (LPL), and heightened blood pressure. The administration of HES resulted in a significant reduction in glucose, HbA1c, LDL, TC, TG, CES, and blood pressure, while concurrently elevating the levels of LCAT and LPL, with no significant alteration observed in HDL-C. These findings demonstrated that HES enhances glycemic regulation, modulates lipid metabolic pathways, and alleviates cardiovascular complications in diabetic models of myocardial infarction, thereby underscoring its potential as a therapeutic agent for the management of myocardial injury in diabetic environments (85). Another study examined the impact of HST on cardiac inflammation and fibrosis subsequent to MI utilizing a murine model. Myocardial infarction was induced by means of left anterior descending coronary artery ligation, while the subjects received treatment with HST (30 mg/kg/day) or a control vehicle over a duration of 8 weeks. Comprehensive evaluations of cardiac function, fibrosis, and inflammation were conducted through echocardiography, histological assessments, and gene expression analyses. HST markedly diminished the levels of inflammatory cytokines (TNF-α, IL-1β, IL-6) and fibrosis indicators (CTGF, collagen I, III), as well as reduced collagen accumulation in the myocardium following MI. From a mechanistic perspective, HST was found to inhibit the activation of the NF-κB signaling pathway, which is a pivotal factor in mediating post-MI inflammation and fibrotic remodeling. These results suggested that HST mitigates cardiac fibrosis and inflammation following MI primarily through the suppression of NF-κB signaling, thereby underscoring its potential utility as a therapeutic agent in the prevention of adverse cardiac remodeling and the progression toward heart failure (80). Overall, naringin, HES, and HST demonstrate pronounced cardioprotective properties against myocardial infarction through the attenuation of oxidative stress, inflammation, apoptosis, and detrimental cardiac remodeling. By engaging in the modulation of critical signaling cascades such as PI3K/Akt/mTOR, NF-κB, and GSK-3β/β-catenin, these flavonoids safeguard myocardial integrity and functionality, underscoring their potential as natural pharmacological agents for the prophylaxis and management of myocardial infarction.

## 7 Future directions and challenges

Despite the extensive empirical evidence corroborating the cardioprotective potential of naringin, HES, and HST, several pivotal obstacles impede their transition from experimental results to clinical implementation. A primary constraint resides in their inadequate oral bioavailability, rapid metabolic degradation, and limited systemic distribution. These flavonoids are subjected to significant first-pass metabolism, culminating in subtherapeutic plasma concentrations that may fail to elicit sufficient biological responses in cardiac tissues. Future research endeavors should consequently prioritize the enhancement of pharmacokinetic characteristics through sophisticated drug delivery systems, encompassing nanoencapsulation, liposomal formulations, polymeric nanoparticles, and phytosome-based carriers. Furthermore, the development of prodrugs or structural analogs may augment both solubility and tissue selectivity, thereby facilitating more reliable cardioprotective effects. Although a multitude of studies has elucidated the modulation of signaling pathways, the comprehensive molecular framework of these compounds’ cardioprotective mechanisms remains inadequately defined. Integrative omics-based methodologies encompassing transcriptomics, proteomics, metabolomics, and lipidomics have the potential to elucidate novel molecular targets and regulatory networks modulated by these flavonoids. Approaches rooted in systems biology and network pharmacology may further elucidate the mechanisms by which multi-target interactions and pathway crosstalk confer cardiovascular protection, thereby facilitating a more comprehensive understanding of their biological effects. Another significant obstacle pertains to the scarcity of translational and clinical investigations. The majority of extant data originate from preclinical models that inadequately represent the intricacies associated with human cardiovascular pathologies. Thus, large-scale, randomized controlled clinical trials are critically required to ascertain the efficacy, optimal dosing, and safety profiles of naringin, HES, and HST within human cohorts. Such trials ought to integrate robust biomarkers indicative of oxidative stress, inflammation, and cardiac remodeling to effectively connect molecular mechanisms with clinical outcomes. Comparative investigations exploring the impacts of individual flavonoids in relation to their synergistic application could offer deeper insights into potential interactive effects. The assessment of safety and long-term efficacy is also imperative. Although these flavonoids derived from citrus fruits are predominantly considered safe, extended or high-dose supplementation may result in unanticipated outcomes. The possibility of interactions with standard cardiovascular medications, such as statins, antiplatelet agents, and beta-blockers, necessitates thorough examination. Extensive toxicological research and pharmacovigilance data are essential to guarantee secure clinical application, especially in geriatric populations and individuals with comorbidities. Moreover, challenges regarding regulatory frameworks and standardization must be resolved prior to the translation of these findings into clinical practice. Variations in the botanical origin, methodologies of extraction, and levels of purity can result in considerable disparities in biological efficacy. The establishment of standardized formulations, validated quality control metrics, and adherence to Good Manufacturing Practice (GMP) will be imperative to ensure reproducibility and safety. Elucidating the regulatory framework pertaining to the approval of flavonoid-based nutraceuticals and phytopharmaceuticals across diverse regions will also constitute a pivotal advancement toward their effective therapeutic application. In conclusion, actualizing the clinical potential of naringin, HES, and HST necessitates a coordinated endeavor that integrates molecular investigation, pharmaceutical innovation, and clinical validation. Surmounting existing challenges through interdisciplinary collaboration will expedite the conversion of these natural compounds into potent cardioprotective agents and contribute to the formulation of safer and more efficient therapeutic approaches for cardiovascular ailments.

## 8 Conclusion

Naringin, HES, and HST, the predominant flavonoids derived from citrus fruits, have surfaced as highly promising bioactive compounds exhibiting significant cardioprotective effects against conditions such as atherosclerosis, cardiac remodeling, and myocardial ischemia/infarction. Comprehensive experimental investigations have indicated that these bioactive substances demonstrate a diverse array of molecular actions, which include antioxidant, anti-inflammatory, anti-apoptotic, lipid-regulating, and endothelial-protective properties. Through the modulation of pivotal signaling pathways, these flavonoids proficiently alleviate oxidative stress, inhibit the release of pro-inflammatory cytokines, hinder the proliferation of vascular smooth muscle cells, and enhance mitochondrial functionality and energy metabolism. Collectively, these intricate mechanisms play a crucial role in preserving myocardial integrity, minimizing infarct size, and improving overall cardiac function. In addition to their direct cardioprotective attributes, these compounds also exert an influence on systemic metabolic and vascular functions that are integral to the pathogenesis of CVDs. By ameliorating lipid profiles, obstructing platelet aggregation, and augmenting the production of endothelial nitric oxide, naringin, HES, and HST collectively facilitate vascular homeostasis and impede the progression of atherogenesis. Furthermore, their capacity to mitigate myocardial fibrosis, hypertrophy, and apoptosis underscores their clinical significance in both ischemic and non-ischemic cardiomyopathies. Notwithstanding these encouraging results, the transference of preclinical observations into clinical practice continues to present considerable obstacles. The limited oral bioavailability, rapid metabolic degradation, and constrained tissue distribution of these flavonoids restrict their pharmacological efficacy. Consequently, forthcoming investigations should prioritize the formulation of optimized delivery systems such as nanoparticle-based strategies or structural analogs to enhance stability, bioavailability, and targeted myocardial accumulation. In addition, extensive clinical trials are imperative to confirm efficacy, establish optimal dosing protocols, and evaluate safety and possible interactions with standard cardiovascular treatments. From a more comprehensive viewpoint, naringin, HES, and HST exemplify a class of naturally occurring cardioprotective compounds capable of simultaneously modulating numerous molecular pathways. Their pleiotropic properties provide a cohesive therapeutic framework that corresponds with contemporary methodologies focused on the prevention and management of multifaceted cardiovascular disorders. Ongoing interdisciplinary investigations that integrate molecular biology, pharmacology, nanotechnology, and clinical medicine will be essential to fully realize their therapeutic efficacy. In summation, these flavonoids derived from citrus fruits exhibit significant potential as supplementary or adjunctive therapies within the realm of cardiovascular medicine. By connecting molecular pathways with clinical implementations, prospective translational studies may position naringin, HES, and HST as integral elements in the formulation of innovative, safe, and efficacious cardioprotective interventions designed to alleviate the worldwide prevalence of CVDs.
